# Supplementary material for: Spin-polarized oxygen evolution reaction under magnetic field
Source: Nat Commun. 2021 May 10;12:2608. doi: 10.1038/s41467-021-22865-y (PMC8110536; doi:10.1038/s41467-021-22865-y)
Supplement: Supplementary file 1 — Supplementary Information [file 41467_2021_22865_MOESM1_ESM.pdf]

Supplementary Materials

Spin-polarized Oxygen Evolution Reaction under Magnetic Field

Xiao Ren[1,2], Tianze Wu[1,2,3], Yuanmiao Sun[2], Yan Li[1], Guoyu Xian[1], Xianhu Liu[4], Chengmin Shen[1], Jose Gracia[5], Hong-Jun Gao[1], Haitao Yang[1]*, Zhichuan J. Xu[2,3,6]*

[1] Beijing National Laboratory for Condensed Matter Physics and Institute of Physics, Chinese Academy of Science, P.O.Box 603, Beijing, 100190, China;

[2] School of Material Science and Engineering, Nanyang Technological University, 50 Nanyang Avenue, Singapore 639798, Singapore;

[3] Solar Fuels Laboratory, Nanyang Technological University, 50 Nanyang Avenue, Singapore 639798, Singapore;

[4] Key Laboratory of Advanced Material Processing & Mold (Zhengzhou University), Ministry of Education, Zhengzhou 450002, China;

[5] MagnetoCat SL, General Polavieja 9 3I, 03012 Alicante, Spain;

[6] Energy Research Institute @ Nanyang Technological University, 50 Nanyang Avenue, Singapore 639798, Singapore.

E-mails: xuzc@ntu.edu.sg; htyang@iphy.ac.cn

Detailed Experimental Methods

Oxides synthesis. Spinel $CoFe_2O_4$ oxides were synthesized by a modified conventional solid-state chemistry method as described elsewhere[1] with $Fe(NO_3)_2$ and $Co(NO_3)_2$ as precursors. 9 mmol mixture of $Fe(NO_3)_3{\cdot}9H_2O$ (Alfa Aesar) and $Co(NO_3)_2{\cdot}6H_2O$ (Sigma Aldrich) was dissolved in 15 mL of DI water, followed by stirring and vaporizing in oven at 80 °C. The resulting slurry was calcinated at 250 °C for 2 hours in the air to decompose nitrous completely. After grinding, the black oxide powders underwent calcination in air at 400 °C for 8 hours. Co_3O_4 oxides were synthesized by the same method.

Electrochemical characterization. The OER tests were operated in a three-electrode cell with a working electrode (WE) of glassy carbon flake (10 mm × 20 mm× 0.5 mm; Effective electrode area: 1.0 cm^2), a counter electrode of platinum foil, and a Hg/HgO reference electrode (RE) (filled with 1M KOH solution). The catalysts electrode was fabricated by the recipe drop-castes method which was reported in elsewhere[2]. The catalysts were mixed with acetylene black (AB) at a mass ration of 5:1, then were dispersed in isopropanol/water (v/v=1:4) solvent followed by the addition of Na^+-exchanged Nafion as the binder. The mixtures were ultrasonicated for 30 min to reach homogeneous ink. The concentration of oxides in ink is 5 mg/ml, and AB is 1 mg/ml. Before drop-casting, the glassy carbon electrodes were polished to a mirror finish with α-Al_2O_3 (50 nm) and washed by IPA and water to clean up completely. Finally, the as-prepared ink (100 ul) was dropped onto glassy carbon electrodes to reach a loading mass of 500 $\mu g_{ox}\ cm^{-2}$ and the electrodes were dried overnight at room temperature. Cyclic voltammograms (CVs), linear sweep voltammetry (LSV) and chronoamperometry (CA) were performed in O_2-saturated 1 M KOH by using Bio-logic SP 150 potentiostat. CA test under the different magnetic field strength (0, 500, 1000, 3000, 5000, 7500, and 10000 Oe) at a constant potential of 1.66 V versus RHE for $CoFe_2O_4$, Co_3O_4, and 1.56V versus RHE for IrO_2. All potentials were converted to RHE scale according to the following equation: $RHE = Hg/HgO + 0.098$ with iR correction. The tests of methanol oxidation reaction (MOR) and ethylene glycol oxidation reaction (EGOR) on CFO electrodes are similar to the OER test. The difference is that the MOR and EGOR were studied in 1M KOH 100 ml electrolyte in the presence of 1 ml methanol and 1ml ethylene glycol, respectively[3].

Materials characterization. The X-ray diffraction (XRD) of oxides were carried on Bruker D8 diffractometer at a scanning rate of 2° min^{-1}, under Cu-K_α radiation (λ = 1.5418 Å). DC magnetization

measurements were performed on a Superconducting Quantum Design (SQUID) magnetometer (MPMS-XL). The SQUID measurements of the magnetization of samples as a function of the magnetic field were carried out at 300 K in fields between -5 T and +5 T. The high-resolution transmission electron microscopy (HRTEM) was carried JEOL JEM- 2100 plus microscope at 200KV. The STEM results presented here were obtained using the 200kV JEOL ARM electron microscope equipped (JEOL, Tokyo, Japan) with double aberration correctors, a dual-energy-loss spectrometer and a cold field emission source. The atomic-resolved STEM images were collected with a condense aperture of 28 mrad and a collection angle of 90 – 370 mrad for HAADF and 11 – 23 mrad for ABF images. The XPS measurements were performed using PHI-5400 equipment with Al Kα beam source (250 W) and a position-sensitive detector (PSD) was used to determine the surface composition of the materials. The Fourier transform infrared spectroscopy–Raman spectroscopy was carried with a confocal Raman microscope (Horiba HR Evolution), equipped with a diode laser emitting at 532 nm. The nominal laser power was filtered down to 1mW to avoid sample overheating. Spectra were recorded with the accumulation time of 60 s. We have carried out HRTEM observation on the $CoFe_2O_4$ catalyst at the identical location before and after the electrochemical reaction using identical location TEM technique (IL-TEM). The detailed methods of IL-TEM are as follows. A diluted ink containing catalyst was pipetted onto the gold finder grid (400 mesh, TED PELLA, USA). The pristine catalyst on the gold finder grid was observed at the specific location before cycling. Then the grid was used at the working electrode in an electrochemical cell. After the electrochemical cycling, the grid was dried under Ar flow and observed under TEM again. The grid allowed us to find the particle at the same location as it was before electrochemical cycling. The images are shown in Supplementary Figure 11. It is clear that those particles remain unchanged after electrochemical cycling and there is no remarkable surface change.

DFT studies. All the density functional theory (DFT) calculations were performed by Vienna Ab-initio Simulation Package[4, 5] (VASP), employing the Projected Augmented Wave[6] (PAW) method. The revised Perdew-Burke-Ernzerhof (RPBE) functional was used to describe the exchange and correlation effects.[7-9] The GGA + U calculations are performed using the model proposed by Dudarev et al.[10], with the U_{eff} (U_{eff} = Coulomb U – exchange J) values of 3.3 eV and 4 eV for Co and Fe, respectively. For all the geometry optimizations, the cutoff energy was set to be 500 eV. A 3×3×1 Monkhorst-Pack grids[11] was used to carry out the surface calculations on the (111) surface of $CoFe_2O_4$. At least 20 Å vacuum layer

was applied in z-direction of the slab models, preventing the vertical interactions between slabs.

In alkaline conditions, OER could occur in the following four elementary steps:

$$OH^- + * \rightarrow *OH + e^- \quad (1)$$

$$OH^- + *OH \rightarrow *O + e^- \quad (2)$$

$$OH^- + *O \rightarrow *OOH + e^- \quad (3)$$

$$OH^- + *OOH \rightarrow * + O_2 + H_2O + e^- \quad (4)$$

where * denotes the active sites on the catalyst surface. Based on the above mechanism, the free energy of three intermediate states, *OH, *O, and *OOH, are important to identify a given material's OER activity. The computational hydrogen electrode (CHE) model[12] was used to address the electrochemical proton-electron transfer with applied potential. The free energies of each elementary step were defined as

$$\Delta G_{ads} = \Delta E_{ads} + \Delta E_{ZPE} - T\Delta S_{ads} \quad (5)$$

where ΔE_{ads} is the electronic adsorption energy, ΔE_{ZPE} is the zero point energy difference between adsorbed and gaseous species, and $T\Delta S_{ads}$ is the corresponding entropy difference between these two states. The electronic binding energy is referenced as ½ H_2 for each H atom, and (H_2O – H_2) for each O atom, plus the energy of the clean slab. The corrections of zero point energy and entropy of the OER intermediates can be found in the Supplementary Table 2. The details of computational under magnetic field are as follows. The function of the outer magnetic field is to align all the randomly oriented spin in the catalyst to a specific direction. To model this situation, we have used the 'LNONCOLLINEAR' and 'SAXIS' keywords to make the spin in the catalyst to a specific direction. And during the calculations, we did not set initial guesses of the magnetic moments and let VASP to fully relax until finding out the most stable configuration. In the Supplementary Table 3, we have summarized the final magnetic moments (μ_B) of the metal cations after structural optimization. The spin densities (μ_B) of the adsorbed oxygen species summarized in Supplementary Table 4.

The surface Pourbaix diagram was calculated based on the method proposed by Hansen et al.[13], where the free energy of oxygen and hydroxyl exchange at a given surface at any pH and potential is calculated as

$$G(HO^*) = \Delta G_0(HO^*) - eU_{SHE} - k_B T \ln 10\, pH + \Delta G_{field} \quad (6)$$

Where ΔG_{field} is the change in the adsorption energy due to the electric field in the electrochemical double layer at the cathode. According to the work by Rossmeisl et al.[14], the relative stability change of O* and OH* under electric field is sufficiently low and it is believed that the trend in adsorption energies can be well described by neglecting ΔG_{field} in the construction of the surface Pourbaix diagram.

The term ΔG_0 is calculated by correcting the DFT energies for zero-point energies and entropy via

$$\Delta G_0 = \Delta E + \Delta ZPE - \mathrm{T}\Delta S \quad (7)$$

Where ΔE and ΔZPE are the DFT energy and the change in zero-point energy of the adsorbates, respectively. More details of the calculation setup can be found in reference.[13]

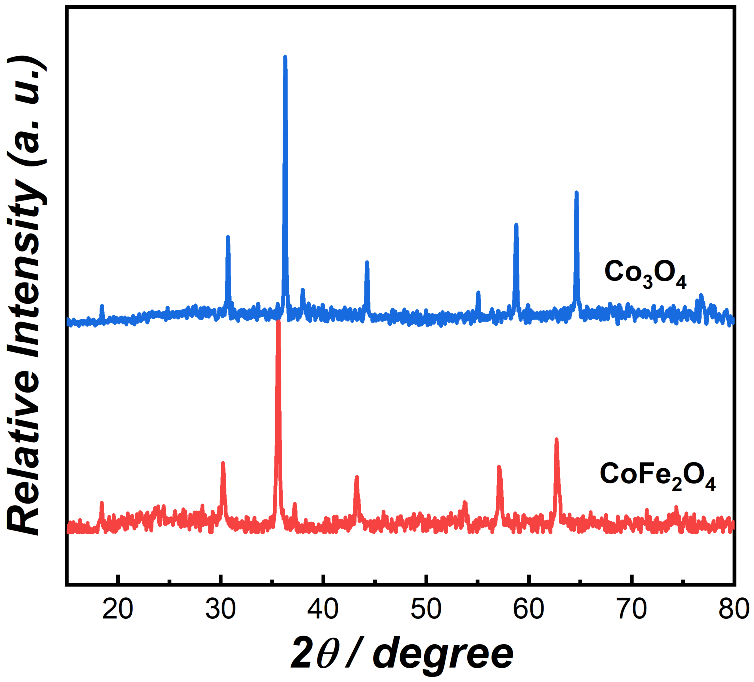

Supplementary Figure 1. The powder X-ray diffraction (XRD) patterns of as-synthesized $CoFe_2O_4$ and Co_3O_4. The details are summarized in Table S1.

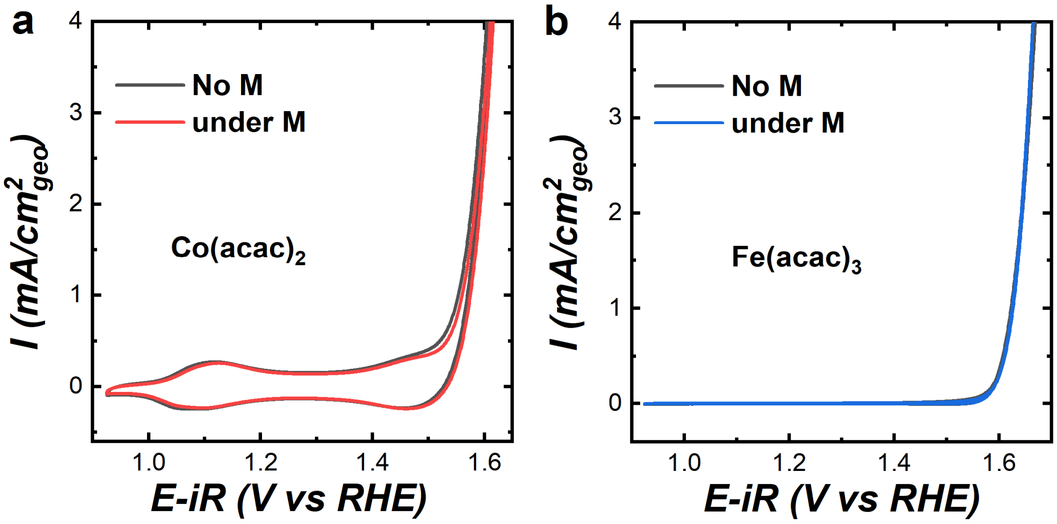

Supplementary Figure 2. Cyclic voltammetry (CV) of $Co(acac)_2$, (**a**) and $Fe(acac)_3$ (**b**) catalysts at a scan rate of 10 mV/s in O_2-saturated 1 M KOH with and without a constant magnetic field (10000 Oe).

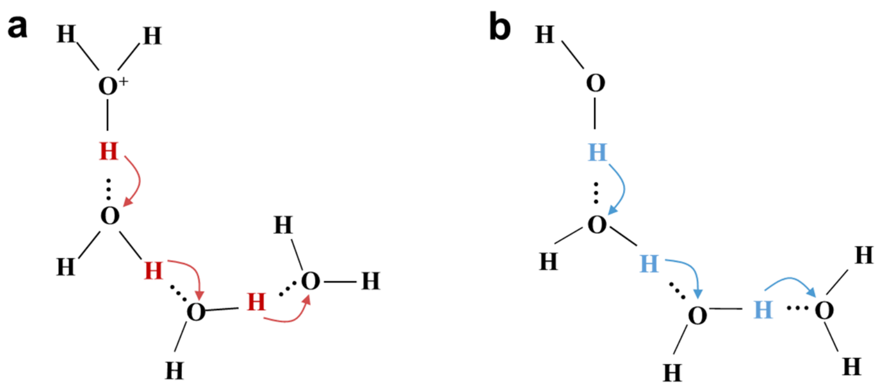

Supplementary Figure 3. The mechanism of proton hopping (jump) for (**a**) H_3O^+ and (**b**) OH^- in aqueous solution. The details of the Grotthuss mechanism can be found in the reference.[15]

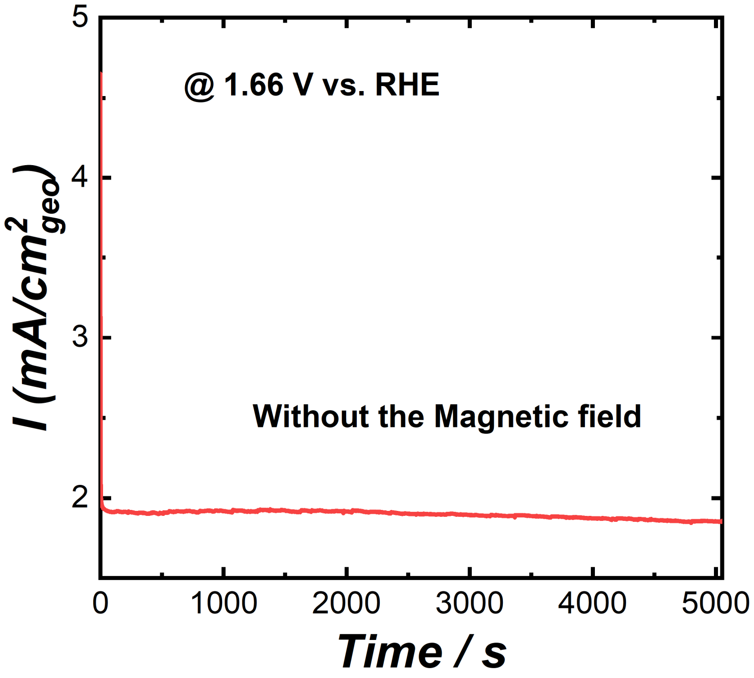

Supplementary Figure 4. CA test in 1 M KOH at a constant potential of 1.66 V versus RHE for $CoFe_2O_4$.

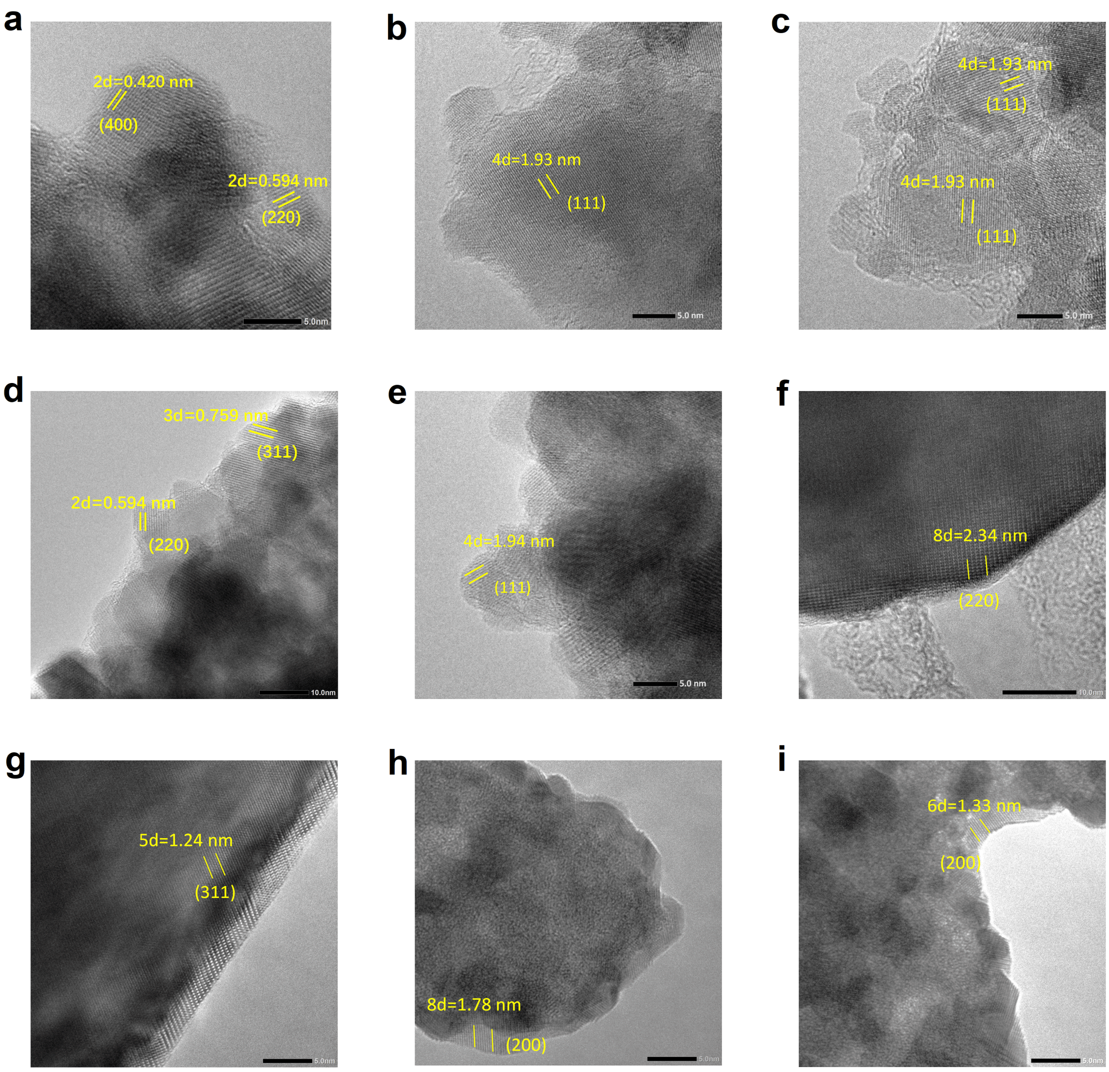

Supplementary Figure 5. HRTEM images of $CoFe_2O_4$ before (**a-c**) and after OER (**d, e**). HRTEM images of Co_3O_4 before (**f**) and after OER (**g**). HRTEM images of IrO_2 before (**h**) and after OER (**i**).

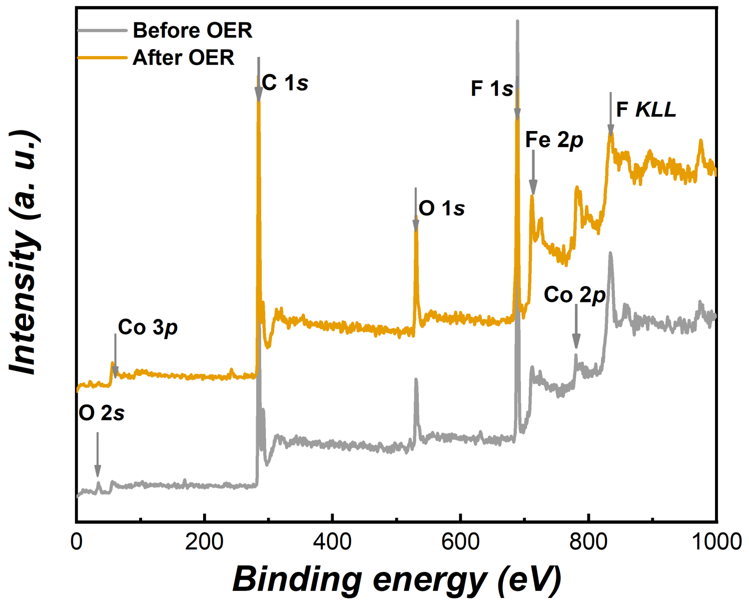

Supplementary Figure 6. The XPS survey spectra of $CoFe_2O_4$ before and after OER.

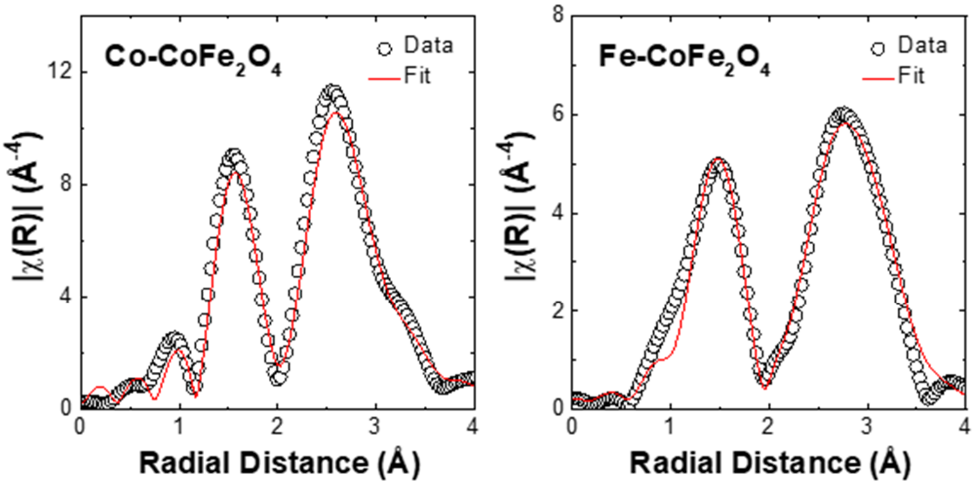

	Tetrahedral site	Octahedral site
Co_x	0.109	0.891
Co-O (Å)	1.81	2.06
Co-O coordination No.	3.3	4.9
Fe_y	0.891	1.109
Fe-O (Å)	1.93	1.99
Fe-O coordination No.	3.0	4.5
χ_{υ}^2 = 376.9; R factor = 0.0188		

Supplementary Figure 7. EXAFS $k^3\chi(R)$ spectra (gray circles) and fitting results (solid lines) of $CoFe_2O_4$ oxides at Co and Fe K-edge. The table show the summary of EXAFS fitting results for $CoFe_2O_4$. It can be seen that $CoFe_2O_4$ cubic spinels, ≈90% Co cations occupy octahedral.

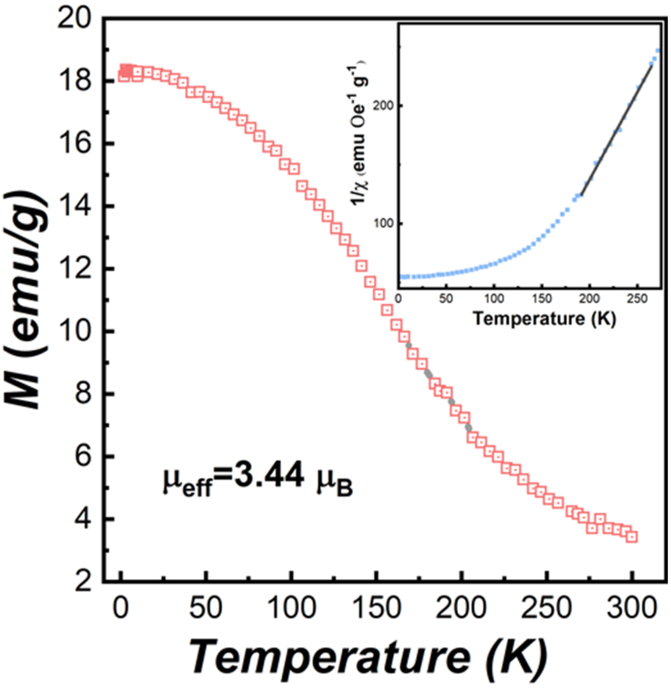

Supplementary Figure 8. The field-cooled M-T curves of $CoFe_2O_4$. The inset figure shows the temperature dependence of reciprocal susceptibilities. The solid line is the fitting results by the Curie–Weiss law. In the high temperature area, the susceptibilities derived from the magnetizations ($\chi = M/H$) obey a Curie−Weiss law: $\chi = C/T - T_C$, where C is Curie constant, and T_C is Curie−Weiss temperature. By fitting the susceptibility versus and T data, an effective magnetic moment μ_{eff} can be obtained through $\mu_{eff} = \sqrt{8C}\ \mu_B$. Here, the calculated μ_{eff} of 3.44 μ_B for the $CoFe_2O_4$ sample is very close to the idea inverse spinel value.

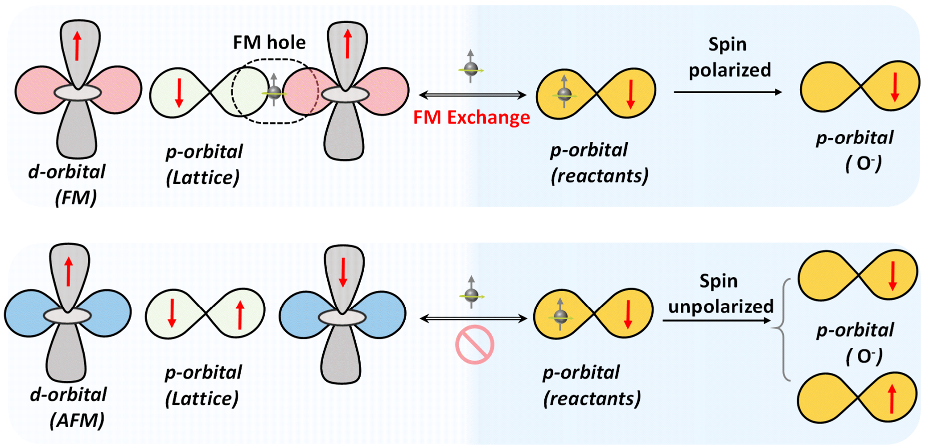

Supplementary Figure 9. Schematic diagram of spin electron transfer in a catalyst. FM holes present in the M−O bonds and also in the oxygen ligands where the dominant FM exchange happens and enhances spin-selective charge transport.

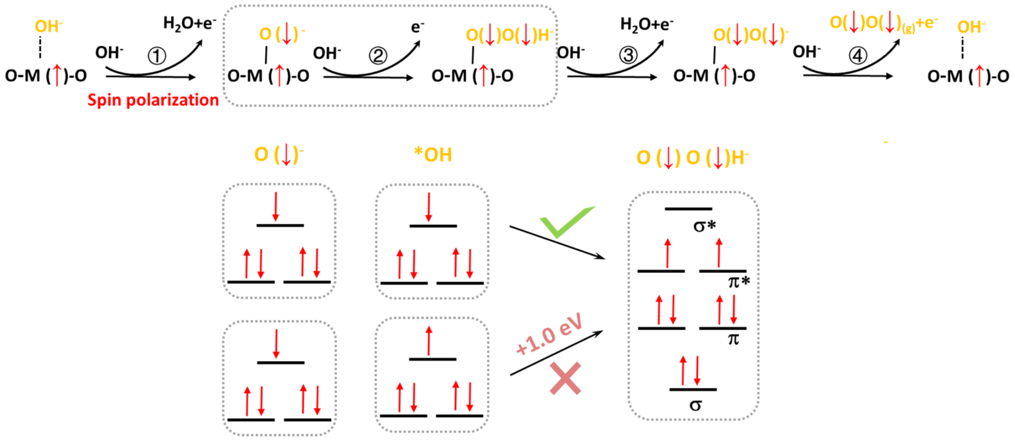

Supplementary Figure 10. The production of the triplet intermediate O(↓)O(↓)H species.

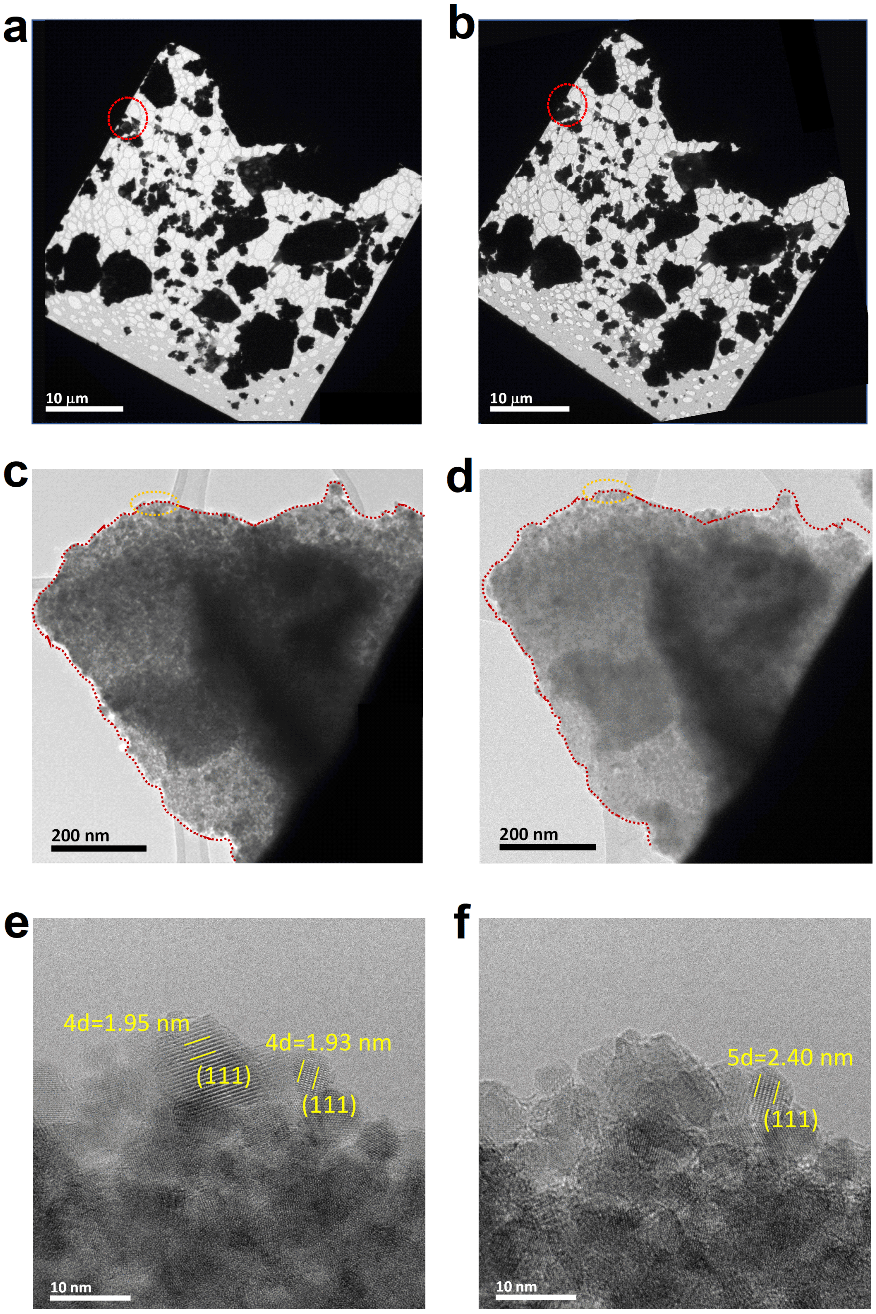

Supplementary Figure 11. Identical location TEM (IL-TEM) images of the $CoFe_2O_4$ before (**a**, **c**, and **e**) and after OER measurement (**b**, **d**, and **f**).

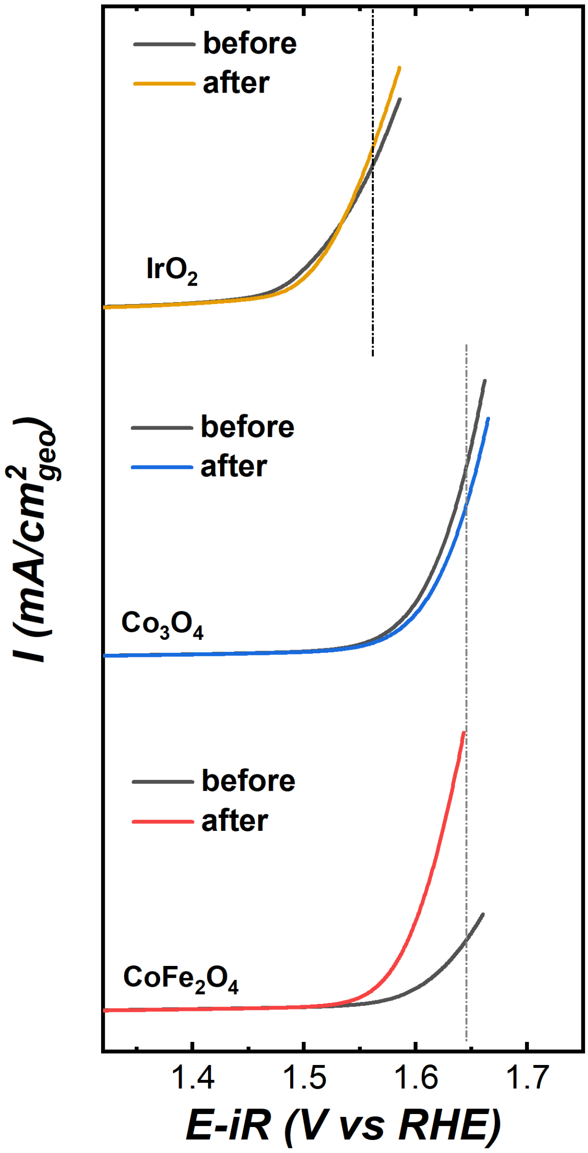

Supplementary Figure 12. Linear sweep voltammetry (LSV) of $CoFe_2O_4$, Co_3O_4, and IrO_2 catalysts at a scan rate of 10 mV/s in O_2-saturated 1 M KOH before and after CA test.

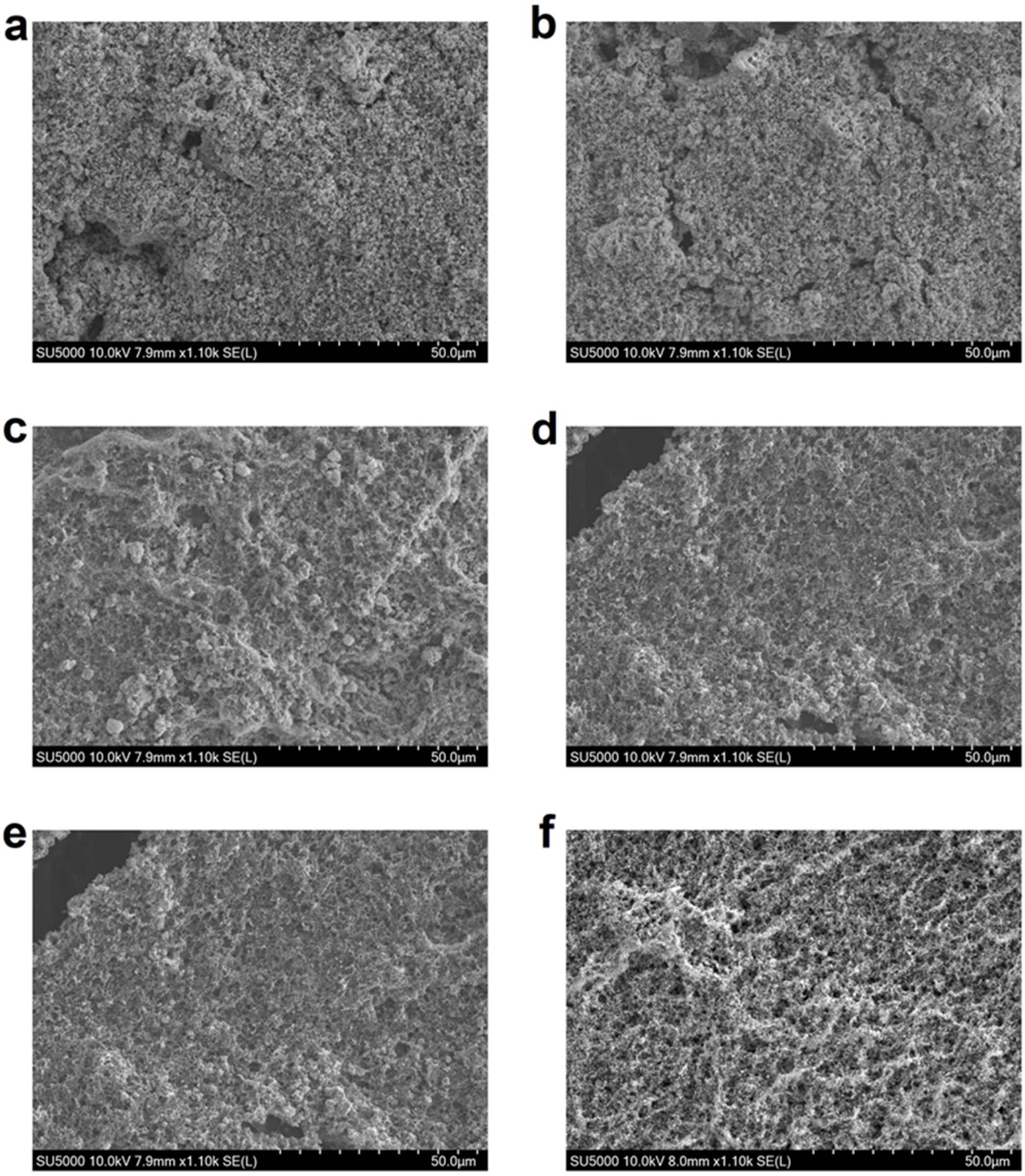

Supplementary Figure 13. SEM images of $CoFe_2O_4$ (**a**) before OER and (**b**) after OER; SEM images of Co_3O_4 (**c**) before OER and (**d**) after OER; SEM images of IrO_2 (**e**) before OER and (**f**) after OER. As shown in Supplementary Figure, the microstructure of $CoFe_2O_4$, Co_3O_4, and IrO_2 before and after OER test under magnetic field have no remarkable difference observed.

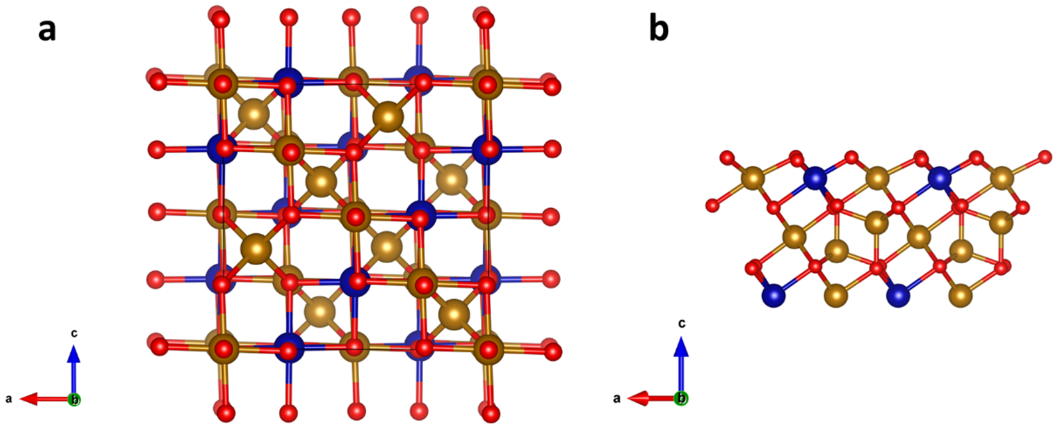

Supplementary Figure 14. The calculation model of $CoFe_2O_4$ with side view for bulk (**a**) and surface (**b**).

Supplementary Table 1. Summary crystal structure parameters and magnetic data.

Samples	Crystal structure	PDF	Magnetism (300K)
$CoFe_2O_4$	Spinel (F-3dm)	22-1086	Saturation magnetization (Ms): 44 emu/gCoercivity (Hc): 887 Oe
Co_3O_4	Spinel (F-3dm)	42-1467	Magnetic susceptibility (χ): 3.07*10-5
IrO_2	Rutile (P42/mnm)	43-1019	χ: 0.51*10-6

Supplementary Table 2. The correction of zero point energy and entropy of the adsorbed and gaseous species.

	**ZPE (eV)**	**TS (eV)**
*OOH	0.35	0
*O	0.05	0
*OH	0.31	0.01
H_2O	0.56	0.67
H_2	0.27	0.41

Supplementary Table 3. The magnetic moments (μ_B) of the metal cations before and after structural optimization.

	Fe $_{(Tet)}$	Fe $_{(Oct)}$	Co $_{(Oct)}$
Without aligned $CoFe_2O_4$	4.224	4.087	0.869
With aligned $CoFe_2O_4$	2.451	2.487	0.530

Supplementary Table 4. Calculated spin densities (μ_B) of the adsorbed oxygen species.

	*OH	*O	*OOH
Without spin alignment	0.065	0.195	O1: -0.025<br>O2: -0.063
With spin alignment	0.053	0.091	O1: -0.026<br>O2: -0.044

References

1. Wu, T. et al. Iron-facilitated dynamic active-site generation on spinel $CoAl_2O_4$ with self-termination of surface reconstruction for water oxidation. *Nature Catalysis* **2**, 763-772 (2019).
2. Suntivich, J., Gasteiger, H.A., Yabuuchi, N. & Shao-Horn, Y. Electrocatalytic Measurement Methodology of Oxide Catalysts Using a Thin-Film Rotating Disk Electrode. *J. Electrochem. Soc.* **157**, B1263-B1268 (2010).
3. Sun, S., Zhou, Y., Hu, B., Zhang, Q. & Xu, Z.J. Ethylene Glycol and Ethanol Oxidation on Spinel Ni-Co Oxides in Alkaline. *J. Electrochem. Soc.* **163**, H99-H104 (2015).
4. Kresse, G. & Furthmüller, J. Efficient iterative schemes for ab initio total-energy calculations using a plane-wave basis set. *Phys. Rev. B* **54**, 11169-11186 (1996).
5. Kresse, G. & Hafner, J. Ab initio molecular dynamics for liquid metals. *Phys. Rev. B* **49**, 14251-14269 (1994).
6. Blöchl, P.E. Projector augmented-wave method. *Phys. Rev. B* **50**, 17953-17979 (1994).
7. Perdew, J.P., Burke, K. & Ernzerhof, M. Generalized Gradient Approximation Made Simple. *Phys. Rev. Lett.* **77**, 3865-3868 (1996).
8. Zhang, Y. & Yang, W. Comment on "Generalized Gradient Approximation Made Simple". *Phys. Rev. Lett.* **80**, 890-890 (1998).
9. Hammer, B., Hansen, L.B. & Nørskov, J.K. Improved adsorption energetics within density-functional theory using revised Perdew-Burke-Ernzerhof functionals. *Phys. Rev. B* **59**, 7413-7421 (1999).
10. Dudarev, S., Botton, G., Savrasov, S., Humphreys, C. & Sutton, A. Electron-energy-loss spectra and the structural stability of nickel oxide: An LSDA+ U study. *Phys. Rev. B* **57**, 1505 (1998).
11. Monkhorst, H.J. & Pack, J.D. Special points for Brillouin-zone integrations. *Phys. Rev. B* **13**, 5188 (1976).
12. Nørskov, J.K. et al. Origin of the overpotential for oxygen reduction at a fuel-cell cathode. *The Journal of Physical Chemistry B* **108**, 17886-17892 (2004).
13. Heine A. Hansen, J.R.a.J.K.N. Surface Pourbaix diagrams and oxygen reduction activity of Pt, Ag and Ni(111) surfaces studied by DFT. *Phys. Chem. Chem. Phys.* **10**, 3722–3730 (2008).
14. Jan Rossmeisl, J.K.N., Christopher D. Taylor, Michael J. Janik, and Matthew Neurock Calculated Phase Diagrams for the Electrochemical Oxidation and Reduction of Water over Pt(111). *J. Phys. Chem. B* **110**, 21833-21839 (2006).
15. Miyake, T. & Rolandi, M. Grotthuss mechanisms: from proton transport in proton wires to bioprotonic devices. *J Phys Condens Matter* **28**, 023001 (2016).
